# Functional groups of rotifers and an exotic species in a tropical shallow lake

**DOI:** 10.1038/s41598-020-71778-1

**Published:** 2020-09-07

**Authors:** Marlene Sofia Arcifa, Bruno Barretto de Souza, Cláudio Simões de Morais-Junior, Cyntia Goulart Corrêa Bruno

**Affiliations:** 1 Departamento de Biologia, Universidade de São Paulo, Av. Bandeirantes 3900, Ribeirão Preto, SP 14040-901 Brazil; 2 Programa de Pós-Graduação em Ecologia e Recursos Naturais (PPGERN), Universidade Federal de São Carlos, Rodovia Washington Luiz s/n, São Carlos, SP 13565-905 Brazil; 3 Instituto Estadual de Florestas, Praça Tubal Vilela 03, Centro, Uberlândia, MG 38400-186 Brazil

**Keywords:** Limnology, Freshwater ecology

## Abstract

In freshwater environments the rotifer group may be divided into microphagous and raptorial species regarding their feeding patterns, and such guilds differently interact with other community components. Here, we analyzed the influence of cladocerans, cyclopoid nauplii, temperature, food resources and an exotic species on rotifer guilds, based on weekly samplings for 1 year. We have identified rotifer species and their trophi types in order to separate them into the raptorial and microphagous functional groups. The ratio raptorial:microphagous rotifers (Guild ratio, GR) was used in interaction analyses with cladocerans, nauplii, temperature, food resources and the exotic species *Kellicottia bostoniensis*. Correlations between total rotifers and food (phytoplankton carbon) and temperature were negative and significant, therefore, these factors did not lead to the increase of rotifer community. On the other hand, microphagous rotifers had opposing relation to cladoceran densities, as GR values showed that they became predominant when cladoceran populations declined. The use of density-based GR was adequate, with similar results compared to biomass-based studies regarding interactions with other organisms. Furthermore, we have found no invasive characteristics for the exotic microphagous rotifer, *Kellicottia bostoniensis*, and it seems to be outcompeted by the native microphagous species.

## Introduction

Rotifers are an important component of plankton in aquatic environments and a link in energy flow^1^. They are more opportunistic organisms than copepods and cladocerans, mainly due to their high reproductive rate^2^ and also by their capacity to inhabit special environments, such as sewage ponds and soda lakes^1^.

Temporal variation of rotifer populations differs in temperate and tropical lakes, as temperature is not a prime factor in the tropics. Biotic factors, such as food, predation, and competition may play a more relevant role for rotifer succession, as well as for other zooplankton groups^3,4^. Temporal fluctuations of rotifer populations may be related to those of their competitors and, therefore, the alternation of raptorial and microphagous functional groups may be a response to competition^5^.

The concept of functional diversity has grown and gained importance in recent years in several fields of ecology, although it is not a new concept^6^. Measuring functional diversity means to achieve the diversity of functional characteristics, which are components of the phenotypes of organisms that influence community processes and define species by their ecological role, i.e. how they interact with the environment and other species^7^. Functional characteristics may be morphological, physiological and phenological, which influence individual growth, reproduction and survival^8^. There are few works devoted to functional groups of zooplankton, as emphasized by Barnett et al.^9^. The food capture mode, size and number of eggs are characteristics that can be used as functional traits to determine the functional diversity of zooplankton community.

Functional diversity in rotifers can be characterized in several ways, considering the adaptation of species to the ecosystem. To define functional groups, the Guild Ratio in number (GR) or biomass (GR′) can be used, which assesses the proportion of raptorial and microphagous species^5,10,11^. However, the GR′ approach requires more specific data, such as individual measurements for biomass calculation or use of length–weight ratios obtained in articles. Features used in defining functional groups depend on research’s objectives. Among morphological features, the trophi is very important in taxonomy, but also useful in the functional group characterization. This pharyngeal and chewing structure, made up of several hard and articulated parts, defines the way rotifers feed, such as grinding, suction, grasping and pumping^1,12^. There are nine known types of trophi—malleate, malleoramate, ramate, uncinate, virgate, forcipate, incudate, cardate, and fulcrate^1^.

Some authors have used a more detailed division, identifying rotifers into six food-functional categories, separating species that feed on tiny particles (< 5 µm) to larger particles (50 µm) and predators^13,14^. Functional diversity may be based on more characters, such as defense mechanisms, presence of lorica and characteristics in food acquisition, which were used for assembling community in different strata of a water body^15^. Other less studied aspects such as dispersion, demographic and physiological characteristics would add value to the knowledge about the functional diversity of rotifers^16^.

Not only interaction with native species but also the introduction of alien ones by human action has an important role on zooplankton community. This anthropogenic action has been growing in ecosystems and drawing attention to their effects for several decades^17^. Not all exotic species, however, are considered invasive, as by definition they must be those that settle in the environment and pose a threat to native populations and the ecosystem^18^. Although there are many characteristics to turn species into invasive, they are not always scientifically proven^19^.

Among exotic species, the rotifer *Kellicottia bostoniensis* was introduced in various parts of the world from North America^18,20–23^. In Brazil, this species has already been found in water bodies from southeast, south and center west regions, but studies in the sub- and tropical zones are scarce, and its distribution may be expanded with increasing research^22^. Likewise, investigations addressing its effect on native communities are also insufficient.

This study addresses several aspects of rotifers in a shallow tropical lake: (a) taxonomic composition and types of trophi; (b) use of Guild Ratio (GR) in the characterization of functional groups; (c) functional groups and their fluctuations throughout the year; (d) interaction with cladocerans and cyclopoid nauplii; (e) relationship between rotifers and the factors food and temperature; (f) temporal variation of the exotic species, *Kellicottia bostoniensis*, its interaction with native species and its evaluation as an invasive species.

The hypotheses for this study are: (1) there is competitive interaction between microphagous rotifers and cladocerans and nauplii, all herbivores; (2) less frequent samplings give comparable results on the interaction between microphagous rotifers and cladocerans; (3) there is competitive interaction between microphagous rotifers and the exotic microphagous *K. bostoniensis*; (4) there is a relationship between rotifers and temperature and food, represented by phytoplankton carbon.

## Material and methods

### Study area

Lake Monte Alegre is located at the campus of the University of São Paulo (21° 10′ 04″ S, 47° 51′ 28″ W), Brazil, at an altitude of 500 m a.s.l. This man-made lake was constructed by damming of Laureano Creek in 1942, which belongs to Pardo River basin. Its dimensions are—7 ha area, 5 m maximum depth and average of 2.9 m. The lake is eutrophic, and circulation pattern is warm discontinuous polymictic. As the gate is not manipulated, water outlet is superficial, the residence time is relatively high for its dimensions (ca. 45 days), it functions as a natural lake. The climate in this region is characterized by two seasons: cool–dry (May–September) and warm–wet (October–April).

### Zooplankton, physical, chemical and biological factors

Zooplankton samplings were carried out weekly for 1 year (2011–2012) in the limnetic zone of Lake Monte Alegre (5 m deep). Three 150 L replicates were collected in each campaign with a suction pump (Jabsco ITT Ind., Mod. 34600-000), which releases 30 L min^−1^, collecting integrated samples in the water column. The water was filtered through 58 µm-meshed plankton net and fixed in formaldehyde at 4% final concentration. The rotifers were counted in three subsamples of 1 mL on a Sedgwick-Rafter chamber at a microscope, keeping the coefficient of variation below 0.20^24^. Cladoceran and nauplii were enumerated in three 1 mL sub samples, taken with a Stempel pipette, on a gridded petri dish at a stereomicroscope, respecting the maximum coefficient of variation of 0.20.

The environmental factors analyzed were temperature and dissolved oxygen (Yellow Springs Inc, mod. 95), resulting in weekly profiles in the water column. The euphotic zone boundary was calculated as Secchi depth (m) × 2.7, according to Cole^25^. To evaluate chlorophyll-*a*, integrated water samples were collected in the euphotic zone with the suction pump and analyzed according to Jeffrey and Humphrey^26^. Chlorophyll concentrations were converted to carbon, assuming that it represents 1.25% of algae dry weight, within the limits reported by Reynolds^27^, and carbon is estimated as 50% of algae dry weight. Carbon concentration was used as a proxy of the available phytoplankton for zooplankton. As no evidence of changes in the degree of lake eutrophication was observed (B. B. Souza et al. in preparation), we did not relate rotifers to nutrient concentrations such as total phosphorus and nitrogen.

### Functional groups

In this study we used groups related to the food capture form of rotifers. These characteristics were determined according to available literature, based on trophi morphology and feeding habits^1,5,12,28^. Ratio calculation Ʃ raptorial species/Ʃ microphagous species (Guild Ratio; GR) was based on Smith et al.^10^, using species densities identified in the functional groups. The difference between guilds is that raptorial species are larger and consume items individually, while microphagous species are filter feeders^1^.

### Data analysis

We have applied Spearman rank correlations to identify possible effects of proposed factors (cladoceran and copepod nauplii densities, temperature, food resources, as well as exotic species density) on rotifer density and GR. For the correlation between the total density of rotifers and the temperature, the Box-Cox transformation was performed on the temperature data; the Box-Cox transformation consists of finding an λ such that the transformed data approaches a normal distribution. For this purpose, the program STATISTICA 8.0-StatSoft software was used and we settled the significance level at *p* < 0.05.

It is paramount that as much a hypothesis is tested as high is the probability of getting the Type I error (false positive). We have performed a great number of statistical tests in this research. Even though they resulted in low *p* values, a Holms-Bonferroni test was used to figure out which tests are not reliable. The test consists in calculating a desired (and reliable) alpha for each statistical test according to the alpha previously postulated (*p* value less than 0.05 in our study). Then, the obtained alpha value (after calculating the statistical tests) must be lesser than or equal to the desired alpha.

## Results

### Physical and chemical factors

The temperature showed a minimum value of 18.2 °C, at the bottom of the lake in the cool season (May–September), and a maximum value of 30.3 °C, at surface in the warm season (October–April) (Table 1). Dissolved oxygen peaked in the warm season, with a maximum of 10.5 mg L^−1^ at surface and a minimum concentration of 0.9 mg L^−1^ at the bottom of the lake, following the thermal stratification characteristic for this season.

**Table 1:** Maximum and minimum values of temperature and dissolved oxygen at two depths, surface and 5 m, from May 2011 to April 2012.

	Cool season		Warm season	
	Max	Min	Max	Min
**Temperature (°C)**				
Surface	25.9	18.9	30.3	25.6
Bottom (5 m)	23.1	18.2	26.3	23.3
**Dissolved oxygen (mg L**^**−1**^**)**				
Surface	9.1	5.9	10.5	5.9
Bottom (5 m)	7.3	1.4	3.9	0.9
Depth of the euphotic zone (m)	5.0	3.5	4.4	2.5

### Species composition, trophic groups, and relationships to abiotic and biotic factors

Rotifers were represented by 29 species during the year, being 11 raptors and 18 microphages (Table 2). Although richness was relatively high, 24 species (83% of the total) were not abundant (> 9% relative abundance). *Ascomorpha saltans* was the most abundant raptorial species (maximum 114 ind. L^−1^), followed by *Polyarthra dolichoptera* (max. 57 ind. L^−1^). Among microphages, *Hexarthra mira* (max. 109 ind. L^−1^), *Keratella americana* (max. 89 ind. L^−1^), and *K. cochlearis* (max. 59 ind. L^−1^) were the most abundant. The exotic microphagous species, *Kellicottia bostoniensis*, reached a maximum of 127 ind. L^−1^, however, its relative abundance (9.2%; Table 2) was lower than that of 4 native species. It is also important to emphasize its high persistence in the samples, with 98% of frequency throughout the study. Similarly, at least 2 of the less abundant species were equally frequent (> 90% frequency).

**Table 2:** Rotifer community composition, the feeding strategies, trophi types, relative abundance and frequency of occurrence.

Species	Feeding strategies	Trophi	Relative abundance (%)	Frequency (%)
*Anuraeopsis navicola* Rousselet, 1911		Malleate	0.9	73
*Ascomorpha saltans* Bartsch, 1,870	R	Virgate	**11.1**	65
*Brachionus angularis* Gosse, 1851		Malleate	0.2	17
*Brachionus mirus* Daday, 1905		Malleate	2.8	100
*Brachionus dolabratus* Harring, 1914		Malleate	3.4	77
*Brachionus falcatus* Zacharias, 1898		Malleate	7.7	81
*Collotheca ornata* (Ehrenberg, 1832)	R	Uncinate	1.0	61
*Collotheca* sp*.*	R	Uncinate	0.5	54
*Colurella adriatica* Ehrenberg, 1831		Malleate	< 0.01	4
*Conochilus coenabasis* (Skorikov, 1914)		Malleoramate	8.2	75
*Conochilus dossuarius* Hudson, 1885		Malleoramate	0.3	33
*Filinia opoliensis* (Zacharias, 1898)		Malleoramate	2.5	63
*Hexarthra mira* (Hudson, 1871)		Malleoramate	**16.1**	98
*Kellicottia bostoniensis* (Rousselet, 1908)		Malleate	**9.2**	98
*Keratella americana* Carlin, 1943		Malleate	**12.7**	100
*Keratella cochlearis* (Gosse, 1851)		Malleate	**9.8**	100
*Keratella tropica* (Apstein,1907)		Malleate	1.4	75
*Lecane leontina* (Turner, 1892)		Malleate	< 0.01	2
*Lecane lunaris* (Ehrenberg, 1832)		Malleate	< 0.01	2
*Lepadella dactiliseta* (Stenroos, 1898)		Malleate	< 0.01	2
*Macrocaetus sericus* (Thorpe, 1893)		Malleate	< 0.01	6
*Polyarthra dolichoptera* Idelson, 1925	R	Virgate	6.6	100
*Pompholyx sulcata* Hudson, 1885	R	Malleoramate	0.2	19
*Synchaeta pectinata* Ehrenberg, 1832	R	Virgate	3.7	40
*Trichocerca cylindrica* (Imhof, 1891)	R	Virgate	1.2	56
*Trichocerca insignis* (Harrick, 1885)	R	Virgate	0.3	60
*Trichocerca longiseta* (Schrank, 1802)	R	Virgate	< 0.01	2
*Trichocerca pusilla* (Jennings, 1903)	R	Virgate	0.2	54
*Trichocerca similis* (Wierzejski, 1893)	R	Virgate	< 0.01	2

Letter R designates raptorial species and the others are microphages. In bold are highlighted the most abundant species (> 9%).

Four types of trophi were recorded, the most common being malleate, characteristic of 14 microphagous species (48% of the total; Table 2). On the other hand, uncinate and virgate types, typical of raptorial species, were recorded in 10 species (34% of the total). The malleoramate type can be found in both microphagous and raptorial species, most frequently in microphages, and it was represented by five species.

**Figure 1:**
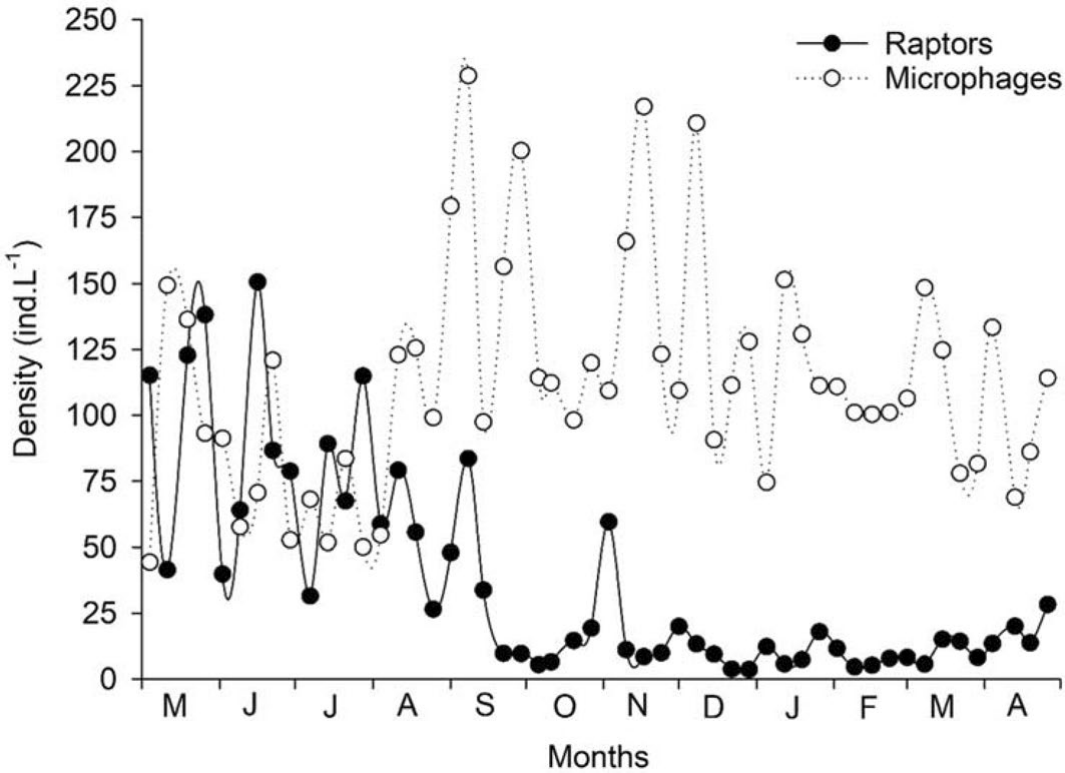
Rotifer densities divided by trophic groups, raptorial and microphagous species, from May 2011 to April 2012.

**Figure 2:**
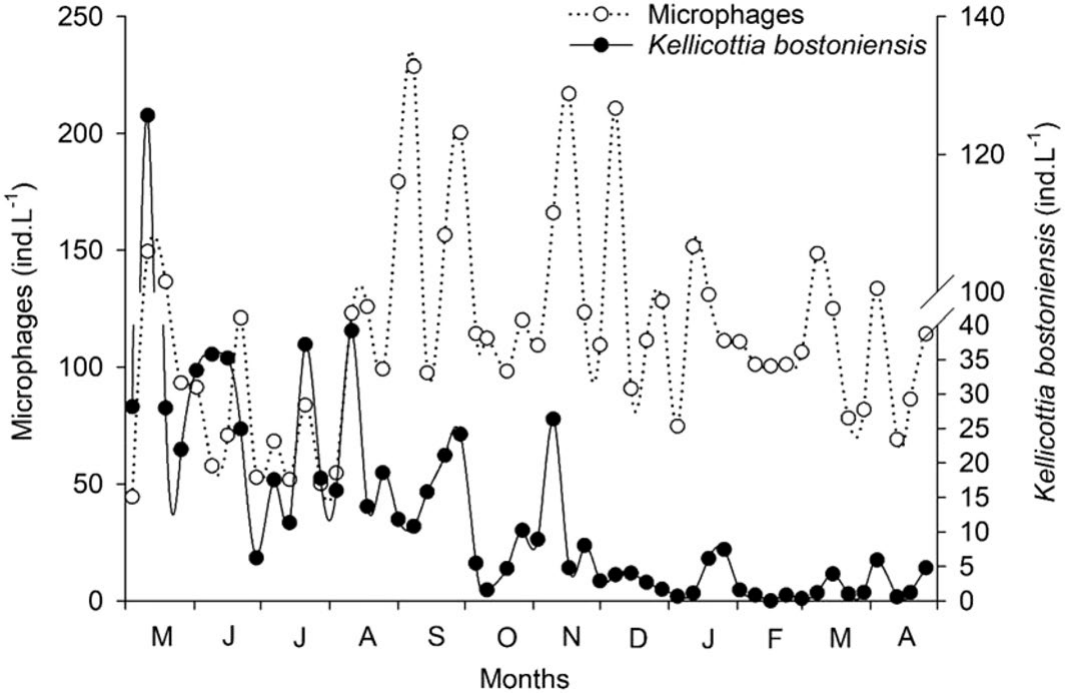
Density fluctuations of the exotic species *Kellicottia bostoniensis* and the other microphagous species, from May 2011 to April 2012.

There was marked fluctuation in rotifer densities for both trophic groups, raptorial and microphagous species, during the year (Fig. 1), with differences between one week and another. At first, both trophic groups oscillated at similar densities, but with alternating peaks. However, from September onward the densities differed, raptorial species decreased from September–October, to values below 25 ind. L^−1^, when the predominance of microphages started, which reached higher densities during this period (up to 225 ind. L^−1^). When microphagous species became dominant, falling tendency for densities of the exotic species *Kellicottia bostoniensis* was observed (Fig. 2), however, there was no significant correlation between them.

**Figure 3:**
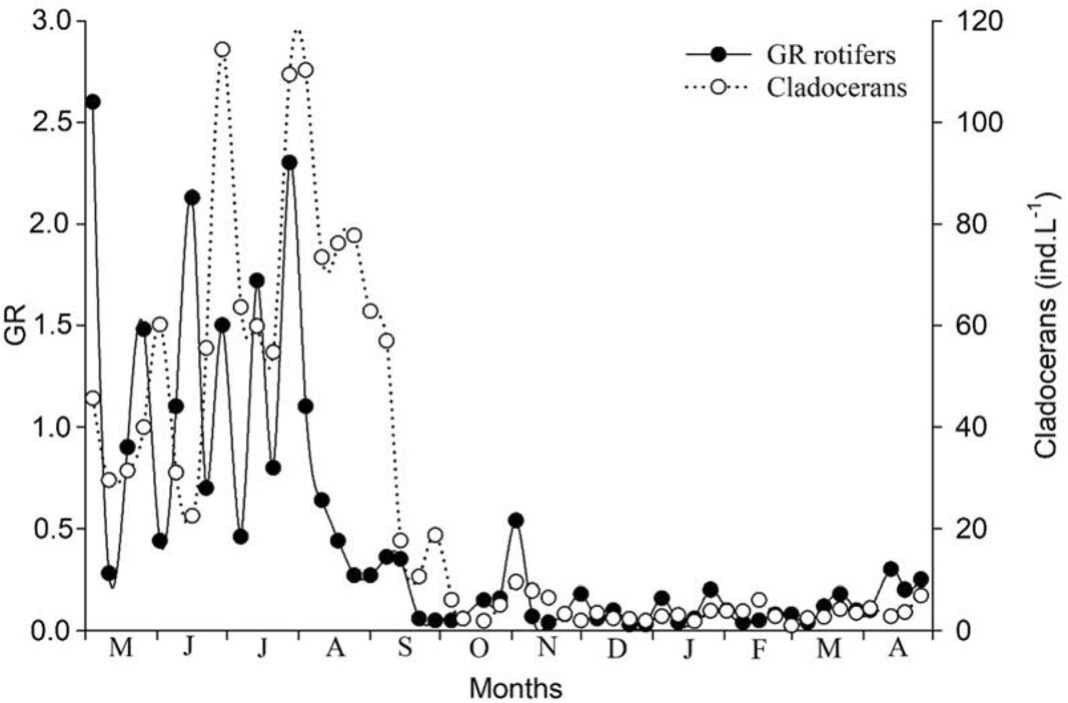
Fluctuations of Guild Ratio (GR) values and cladoceran densities, from May 2011 to April 2012.

**Figure 4:**
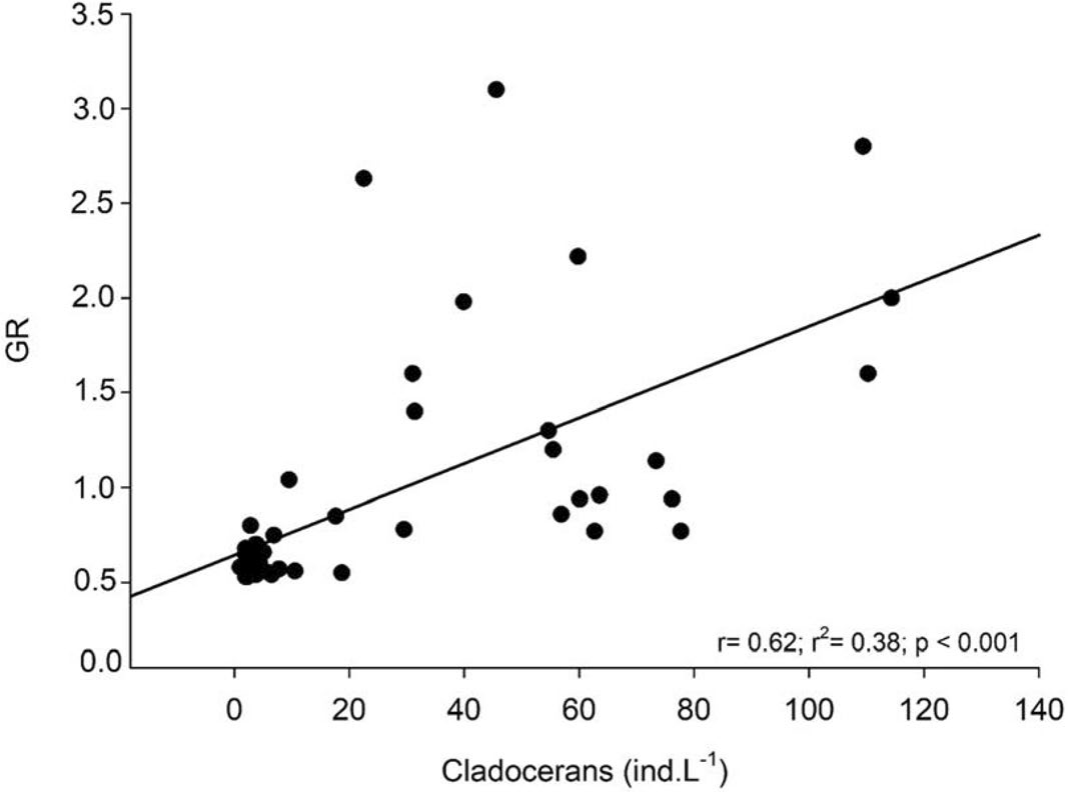
Correlation between GR and cladoceran densities with monthly data.

**Figure 5:**
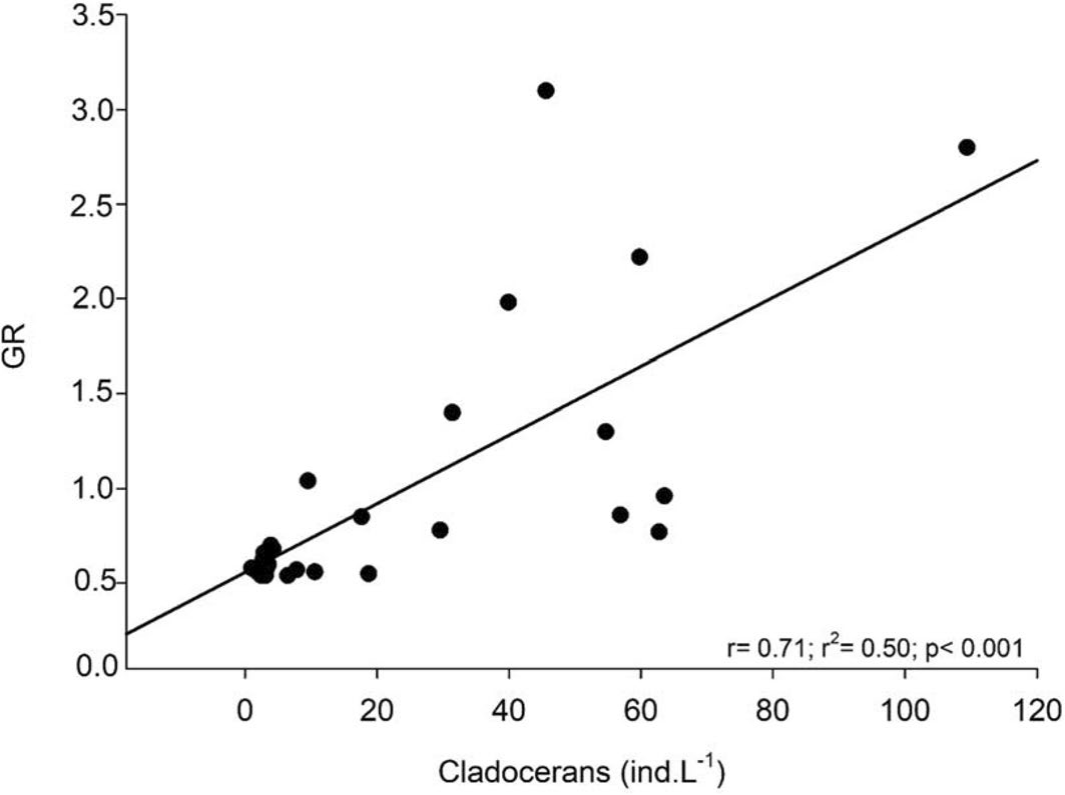
Correlation between GR and cladoceran densities with bimonthly data.

The lowest Guild Ratio (GR) values were recorded when cladoceran densities decreased, from the end of September 2011 until April 2012 (Fig. 3). Correlation between GR and cladoceran densities was positive and significant (r = 0.62, *p* < 0.001; Fig. 4), showing a decrease in GR values, that is, a decrease in raptorial rotifers and a predominance of microphagous rotifers when cladocerans declined from late September 2011 to April 2012. To assess whether less frequent samplings give similar results, we used bimonthly GR and cladoceran density data and the trend was the same as that found with monthly data, with a positive correlation between both factors (r = 0.71; *p* < 0.001; Fig. 5).

**Figure 6:**
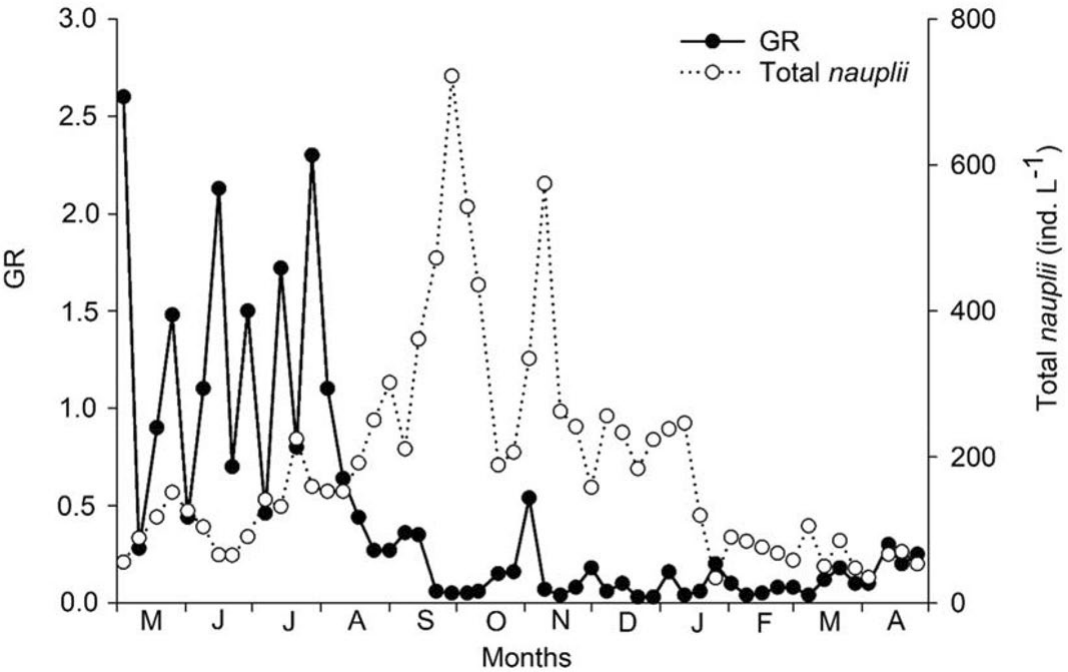
Fluctuations of GR values and nauplii densities, from May 2011 to April 2012.

GR values decreased when the densities of cyclopoid nauplii increased (Fig. 6), and the correlation between them was negative and marginally significant (r =  − 0.25, *p* = 0.06). The dominance phase of microphagous species, indicated by lower GR values, coincided with higher densities of nauplii.

**Figure 7:**
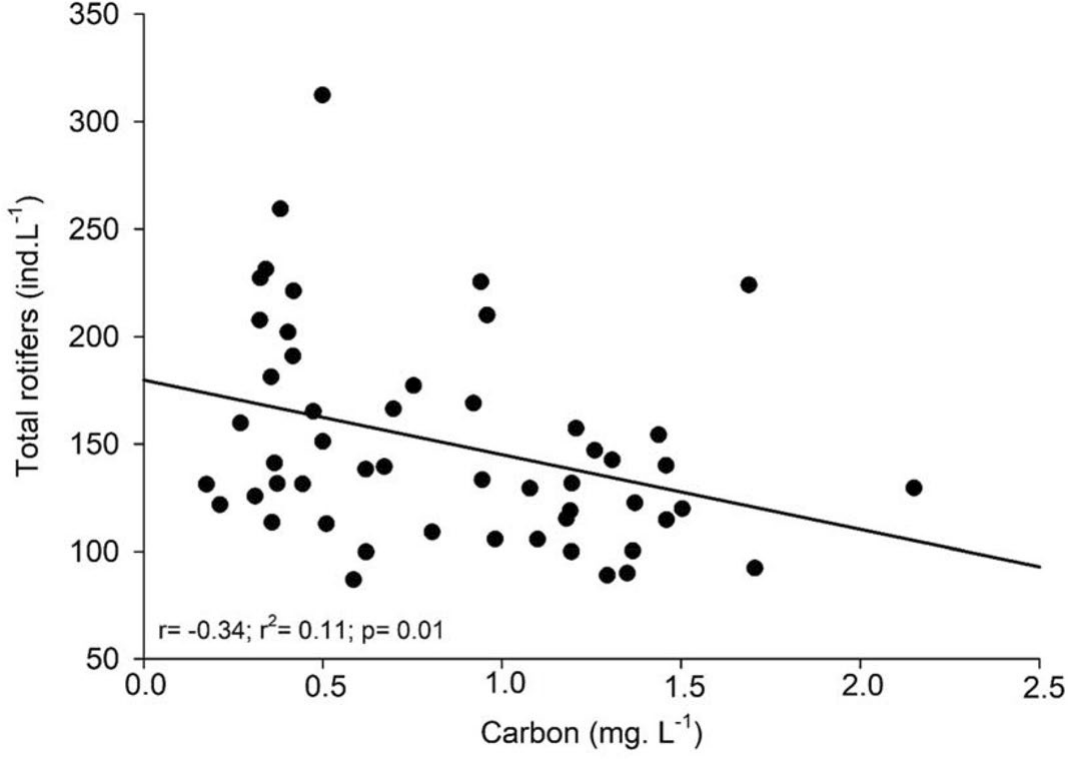
Correlation between total rotifers and phytoplankton carbon concentrations.

**Figure 8:**
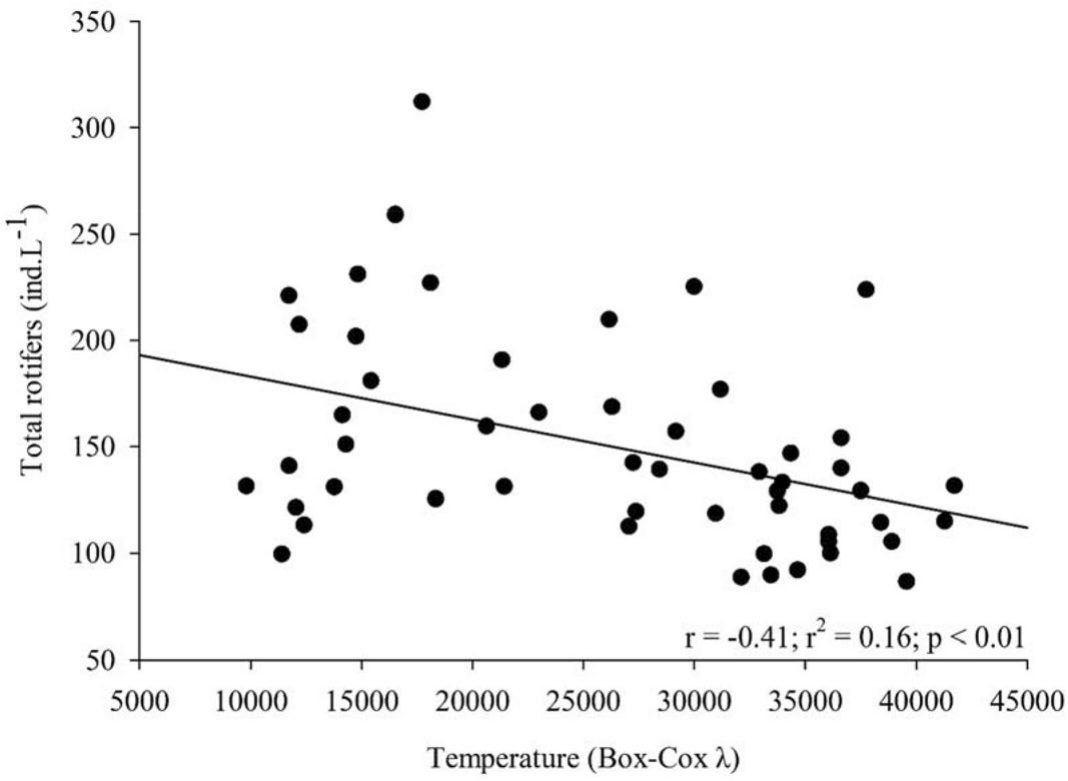
Correlation between total rotifers and temperature.

Regarding resources, we found significant negative correlation between total rotifer densities and carbon concentrations during the study period (r =  − 0.34, *p* = 0.01; Fig. 7) and temperature (r =  − 0.41; *p* = 0.002; Fig. 8). Likewise, relationships between GR and both carbon and temperature also resulted in significant negative correlations (r =  − 0.55, *p* < 0.001; r =  − 0.69, *p* < 0.001, respectively). However, when the correlation effect is restricted only between microphages and carbon, no significant outcome is shown.

## Discussion

Our study on a shallow tropical lake identified fluctuations and interactions of rotifer assemblage, based on trophic guild analysis, comparable to those found in temperate lakes. We also highlighted that trophic guilds, based on trophi structure, has broad application to both temperate and tropical water bodies, which shows the universality of this approach. In addition, our analysis on the interaction between the exotic species *Kellicottia bostoniensis* and other microphagous rotifers were sufficient to demonstrate that it does not have invasive characteristics.

The Guild Ratio (GR), based on the density of raptorial and microphagous functional groups of rotifers, revealed to be an appropriate tool in the evaluation of possible interactions with other planktonic groups, as well as in the evaluation of temporal changes of functional groups. Unlike Obertegger and Manca^5^ and Obertegger et al.^11^, we used the database of densities of functional groups instead of biomass, according to Smith et al.^10^. The significant correlation between GR and cladocerans showed that GR, based on number of individuals, indicated interaction between microphagous rotifers and cladocerans like that reported by Obertegger and Manca^5^ and Obertegger et al.^11^ in temperate lakes, based on biomass (GR′). The relationship GR-cladocerans showed a similar trend with monthly and bimonthly data, indicating its adequacy even when data are less frequently obtained, which agrees with results from Obertegger et al.^11^ in Lake Washington, USA. Given this point, our findings reinforce that other studies may be designed with lower sampling frequency and certainly achieve satisfactory results, allowing a cheaper logistic planning in further research.

The significant positive correlation between GR and cladoceran densities indicates competition between the groups, corroborating the initial hypothesis. The predominance of microphagous rotifers (i.e. lower GR values) when cladoceran densities decreased, represented mainly by *Daphnia gessneri* (max. size 1.22 mm) and *Ceriodaphnia richardi* (max. 0.70 mm)^29^, is a sign of competition between both groups. Therefore, when cladocerans were more abundant during the cool season (May–September), the raptorial rotifers species predominated, which coexist with filtering cladocerans, similarly to results obtained by Obertegger et al.^11^, in lakes Washington (USA) and Caldonazzo (Italy). Exploitative competition between cladocerans and rotifers, particularly microphagous species, which occupy a similar niche, may even lead to competitive exclusion of rotifers. Herbivorous cyclopoid nauplii could compete with rotifers and make our analysis meaningful, however there was no evidence of interaction between them and microphagous rotifers, which does not support our hypothesis.

Several studies report the competitive superiority of cladocerans^4,30,31^. The inferiority of rotifers may be partly due to lower clearance rate (1–10 µL ind.^−1^ h^−1^) than cladocerans (10–150 µL ind.^−1^ h^−1^) as well as a more limited size food range (ca. 4–17 µm)^1^. The maximum clearance rate of cladocerans may be much higher than that already mentioned by Nogrady et al.^1^ and dependent on various factors such as temperature, food concentration and body size^9^. Rotifer populations may be suppressed by more efficient cladocerans through exploitative competition, although rotifers may also suffer effects from interference competition^32,33^. Cladocerans larger than 1.2 mm may suppress small rotifer populations by interference^34^. In Lake Monte Alegre, cladoceran species are relatively small and probably exploitative competition is the most important interaction in this community.

The increase in algal carbon and temperature in the Lake Monte Alegre during the warm season (October–April) was not followed by increase of the total rotifer densities, indicating a preponderant influence of another factor. However, as mentioned above, there was an increase in the abundance of microphagous species and a decrease in densities of raptorial species in this season. Raptorial species, particularly large species (e.g., *Synchaeta* spp.), prefer larger items (> 50 µm) such as algae, ciliates and other rotifers^13^. Species of the genus *Ascomorpha* feed on dinophytes, such as *Peridinium* and *Ceratium*, which are grasped, and the content sucked^1^. In Lake Monte Alegre, an increase of *Peridinium* in the fall and winter (March–September) was already reported^35,36^, which would benefit some raptorial rotifers, including *Ascomorpha*. However, in this study in 2011–2012, dinophytes were not abundant (L.H.S. Silva, unpublished data), representing about 1.4% of the total phytoplankton density, chlorophytes predominating, increasing the contribution of cyanobacteria in the warm season. Therefore, higher densities of raptorial species in the cool season were unrelated to phytoplankton composition and, on the other hand, higher temperatures in the warm season did not favor the increase in populations of this group.

The distribution of organisms can be a strategy to avoid competition and predation. In Lake Monte Alegre, several species of *Colotheca*, *Keratella*, *Polyarthra*, and *Trichocerca* occupied the entire water column in the cool season (A. J. Meschiatti et al. unpublished data). In the warm season, species of these genera, in addition to *Brachionus*, *Hexarthra* and *Ptygura* were limited to the oxygenated layer, avoiding the anoxic hypolimnion. Another feature of the vertical distribution of rotifers in this lake was the frequent occupation of the most superficial layer, even during the day, which is rarely occupied by cladocerans^37^, reducing overlap and possible interactions with other organisms.

Direct predation on rotifers by chaoborid larvae is low in Lake Monte Alegre, representing 9% of the prey number for instars I and II, 4% for instar III, not being preyed on by instar IV^38^. In an experiment with mesocosm in this lake, no predation effect by *Chaoborus* larvae on *Keratella* spp. densities was detected^39^. Zooplankton predation by fish in the lake is mainly exerted by adult of the exotic cichlid *Tilapia rendalli* (current name *Coptodon rendalli*), a pump filter-feeder^40^, which collects organisms with lower evasion to the filtering current, which, however, are not abundant in the lake. Although *Keratella* sp. was not rejected by tilápia, its consumption is low by this fish species, whose predation is higher on cladocerans^40^.

Temporal variations of functional groups of rotifers in Lake Monte Alegre indicated the indirect effect of cladoceran predation by invertebrates, such as *Chaoborus brasiliensis* larvae and the aquatic mite, *Krendowskia* sp., in 2011–2012^41^. Predation pressure by invertebrates is generally higher in the warm season when their populations increased, resulting in declining cladoceran populations^29,41^. Consequently, there is a decrease in exploitative competition by cladocerans and the possibility of competitive exclusion when resources are limiting. Predation by invertebrates has emerged as the main structuring factor of the lake zooplankton^29^, and this study highlights the indirect effect of this factor on rotifers.

The high frequency of occurrence of the exotic species *Kellicottia bostoniensis* in the present study, combined with the weekly sampling strategy adopted, demonstrates the great persistence capacity of this species in the environment, with rare occasions when it is excluded from the water column. This feature indicates success of the exotic species in the new habitat^22^. The characteristics of an invasive species are not always scientifically proven, and many failures are not reported in publications, introducing a bias in evaluating the success of exotic species^19^. The presence of this exotic species in Lake Monte Alegre had not been detected in previous studies conducted in the 1980s^42,43^. Although very common, according to Josefsson and Andersson^18^ it is not invasive in the lake, as it did not constitute a threat to the local community of rotifers. It does not outcompete other microphagous rotifers and, on the contrary, there is evidence of being competitively inferior, as its population decreased in periods of dominance of other microphagous species. A laboratory experiment showed that *K. bostoniensis* had no effect on zooplankton composed of native copepods, cladocerans, and rotifers, affecting only ciliates, which are part of its food resources^23^, reinforcing the idea that it does not constitute a threat to the whole planktonic community.

This exotic species was caught in lakes from River Doce valley^44^ and in Furnas Reservoir^45^, in Brazil, at lower densities than those of Lake Monte Alegre (max. 127 ind. L^−1^). The vertical distribution in Nado Reservoir, located in Brazil, showed its highest abundance in the anoxic hypolimnion, on a diel cycle^46^, indicating resistance of this species to adverse conditions. In some Swedish lakes, the exotic species *K. bostoniensis* was also found in deeper layers^18^, as well as in Mirror Lake, United States, where the production of *K. bostoniensis*, a native species, was higher at the bottom^47^. Apparently, this species maintains similar distribution in its original habitat and a new habitat. The ability to occupy lower layers, often anoxic, where few microcrustaceans and rotifers are found, would lower negative interactions with other populations and even constitutes a defense strategy against predation by most invertebrates and filtering fish^48^.

## Concluding remarks

The use of functional groups in the analysis of temporal variations of rotifers in Lake Monte Alegre proved to be valid for understanding the relationships between populations and interactions with other groups. The density-based Guild Ratio (GR) was adequate in this analysis, and because it is more easily obtained than the biomass-based Guild Ratio (GR′), we suggest that it can be used with confidence. GR showed interaction with cladocerans in weekly or bimonthly collections, i.e., samplings do not need to be highly frequent to obtain a relationship between the factors.

The main factor that would explain the variations of rotifer functional groups in the lake is the interaction with cladocerans, which resulted in the dominance of microphagous species when cladocerans declined due to invertebrate predation, indicating an indirect predation effect on rotifers. Inverse relationship of rotifers with carbon showed that food increase in the warm season did not lead to the increase of the total abundance. Increasing temperature also had no effect on rotifer abundance and rotifer functional groups. At last, the exotic species *Kellicottia bostoniensis* is not an invasive species and even seems to be outcompeted by native microphagous species.
